# MPO-ANCA-associated vasculitis in the context of autoimmune polyglandular syndrome type 3: case report and literature review

**DOI:** 10.1093/rap/rkac085

**Published:** 2022-10-25

**Authors:** Cédric Dikovec, Kevin Wolters, Liv M Vossen, Sören A Gärtner, Rémy L M Mostard, César Magro-Checa

**Affiliations:** Department of Rheumatology, Zuyderland Medical Centre, Heerlen/Sittard-Geleen, The Netherlands; Department of Internal Medicine, Section of Nephrology, Zuyderland Medical Centre, Heerlen/Sittard-Geleen, The Netherlands; Department of Pulmonology, Zuyderland Medical Centre, Heerlen/Sittard-Geleen, The Netherlands; Department of Internal Medicine, Section of Nephrology, Zuyderland Medical Centre, Heerlen/Sittard-Geleen, The Netherlands; Department of Internal Medicine, Section of Nephrology, Zuyderland Medical Centre, Heerlen/Sittard-Geleen, The Netherlands; Department of Pulmonology, Zuyderland Medical Centre, Heerlen/Sittard-Geleen, The Netherlands; Department of Rheumatology, Zuyderland Medical Centre, Heerlen/Sittard-Geleen, The Netherlands

Key message

- MPO–ANCA-associated vasculitis is a potential presentation in the context of autoimmune polyglandular syndrome type 3.

Dear Editor, A 55-year-old White man was admitted to the nephrology department with renal insufficiency, haematuria, progressive fatigue, dry cough and exertional dyspnoea. He had been suffering from general malaise and generalized arthralgias without clear signs of inflammatory arthritis for the last 6 months. His past medical history was remarkable for vitiligo, RP, hypothyroidism owing to Hashimoto’s thyroiditis with elevated thyroid peroxidase antibodies since 2011, and chronic atrophic gastritis with positive intrinsic factor and parietal cell antibodies since April 2021. He was currently treated with levothyroxine, simvastatin, carbasalate calcium, ticagrelor and metoprolol.

At admission, the vital signs were as follows: temperature 38.5°C, blood pressure 94/73 mmHg, regular pulse 81/min, respiratory rate 15/min and oxygen saturation 96% without supplemental oxygen. Physical examination was significant for bilateral fine crackles on pulmonary examination. The skin was remarkable for vitiligo on the lower limbs and thorax; no signs of cutaneous vasculitis or SSc were observed. He had no muscle weakness or inflammatory arthritis. Laboratory testing at admission revealed the following results (normal values in parentheses): CRP 153 (<10) mg/l, ESR 108 (1–20) mm/h, creatinine 199 (61–113) µmol/l, estimated glomerular filtration rate 32 (>90) ml/min/1.73 m^2^, haemoglobin 5.9 (8.5–11.0) mmol/l; urinalysis revealed 463 (<10) erythrocytes/µl with 20–30% dysmorphic erythrocytes and no erythrocyte or granular casts, 76 (<20) leucocytes/µl and mild 24-h proteinuria of 0.68 g. Blood and urine cultures were negative. A chest CT scan showed centrilobular opacities primarily in the right lung and mediastinal lymphadenopathy without signs of cavitary lesions. A bronchoalveolar lavage revealed endobronchial blood without an active bleeding focus. Bronchoalveolar lavage fluid analysis was consistent with diffuse alveolar haemorrhage, showing 10.00 × 10^9^ erythrocytes/l, 721.00 × 10^6^ leucocytes/l with 93% macrophages, and a marked positive iron staining (97% of all macrophages); bacterial, mycobacterial, fungal and viral tests and cultures were negative. Additional serological testing was positive for antibodies direct against MPO [39 (<3.5) IU/ml]. A renal biopsy showed pauci-immune necrotizing crescentic glomerulonephritis. Out of 18 glomeruli, eight were normal, nine showed fibrinoid necrosis (with extracapillary proliferation and/or crescent formation) and one showed segmental sclerosis without any signs of activity. No significant tubular atrophy or interstitial fibrosis was observed. Vessel wall necrosis was observed in one artery. The glomerulonephritis was classified as crescentic type according to the Berden classification and was given a score of zero on both the Mayo Clinic Chronicity Score and the ANCA Renal Risk Score [1]. A diagnosis of MPO–ANCA-associated vasculitis (MPO-AAV) with glomerulonephritis and diffuse alveolar haemorrhage associated with autoimmune polyglandular syndrome type 3 was made. Our patient received three pulses of i.v. methylprednisolone 1000 mg, followed by oral prednisolone 60 mg daily in a tapering dose and rituximab 1000 mg on days 0 and 14, with rapid clinical improvement of the pulmonary symptoms and stabilization of the renal function. Six months after diagnosis, the patient reported no complaints, the MPO-ANCA titre had decreased to 6.5 IU/ml and the estimated glomerular filtration rate was stable at 36 ml/min/1.73 m^2^. Furthermore, prednisolone was tapered down to 5 mg daily and he received one infusion of rituximab 1000 mg as maintenance therapy.

Autoimmune polyglandular syndrome is a heterogeneous group of diseases characterized by immune-mediated activity against endocrine and non-endocrine organs. Autoimmune polyglandular syndrome can be classified into four different subtypes (types 1–4) based on clinical criteria. Autoimmune polyglandular syndrome type 3 includes autoimmune thyroid diseases plus another autoimmune disorder in the absence of Addison’s disease. If the other autoimmune disorder present is an endocrine disease, most commonly type 1 diabetes mellitus, it is designated as type 3A. Type 3B involves gastrointestinal diseases, mostly chronic atrophic gastritis and pernicious anaemia, and type 3C involves cutaneous, neurological and haematological diseases. Type 3D involves systemic autoimmune rheumatic diseases, with SS, RA and SLE being the most frequently reported; other systemic autoimmune rheumatic diseases have been described less frequently [2]. By reviewing the literature, we identified another four cases of AAV in the context of autoimmune polyglandular syndrome (Table 1) [3–6]. Interestingly, four of the five patients presented a similar serotype (MPO–ANCA positive) and four of the five patients had biopsy-proven pauci-immune crescentic glomerulonephritis. Furthermore, all five patients had a history of Hashimoto’s thyroiditis. Autoimmune thyroid diseases are more common in patients with AAV, especially in MPO–ANCA-positive patients and patients with renal disease, than in the general population. This association is potentially attributable to 44% sequence homology between MPO and thyroid peroxidase, resulting in cross-reactivity, or general loss of tolerance to peroxidases [7].

**Table 1.** Summary of reported cases of patients with ANCA-associated vasculitis and autoimmune polyglandular syndrome

Publication	Sex/age (years)	ANCA type	Vasculitis manifestations and signs/symptoms	Treatment	Associated autoimmune diseases	**Autoimmune polyglandular syndrome type** ^a^
Bonomini *et al.* (1998) [3]	F/56	cANCA	General: malaise, nausea, weight loss, weakness Musculoskeletal: arthralgia Renal: pauci-immune crescentic glomerulonephritis Neurological: asymmetric axonal sensorimotor neuropathy	Prednisone^b^ CYC^b^	Hashimoto’s thyroiditis Chronic atrophic gastritis cANCA-associated vasculitis (MPA)	3
Murray *et al.* (2013) [4]	F/42	MPO–ANCA, pANCA	General: not reported Musculoskeletal: arthralgia Renal: pauci-immune crescentic glomerulonephritis	Plasma exchange Prednisolone^b^ CYC^b^ AZA (as maintenance therapy after CYC)^b^	Hashimoto’s thyroiditis Vitiligo Type 1 diabetes mellitus Addison’s disease MPO–ANCA-associated vasculitis	2
Mosakowska *et al.* (2016) [5]	F/75	MPO–ANCA, pANCA	General: malaise, loss of appetite, weakness Renal^c^: renal failure, microhaematuria, proteinuria, decreased diuresis, oedema, hypertension Respiratory: exertional dyspnoea	Methylprednisolone 500 mg i.v. for 3 days followed by prednisone 30 mg in a tapering dose CYC 750 mg i.v. every 3-4 weeks (total dose 4.5 g) AZA 100 mg (as maintenance therapy after CYC)	Hashimoto’s thyroiditis Vitiligo Pernicious anaemia Type 1 diabetes mellitus MPO–ANCA-associated vasculitis	3
Tian *et al.* (2020) [6]	F/51	MPO–ANCA, pANCA	General: not reported Renal: crescentic glomerulonephritis with renal vasculitis	Methylprednisolone 100 mg i.v.^b^	Hashimoto’s thyroiditis Hyperparathyroidism Alopecia Adult-onset Still’s disease MPO–ANCA-mediated crescentic glomerulonephritis with renal vasculitis	3C
Our case	M/55	MPO–ANCA	General: fatigue, malaise Musculoskeletal: arthralgia Renal: pauci-immune crescentic glomerulonephritis Respiratory: diffuse alveolar haemorrhage	Methylprednisolone 1000 mg i.v. for 3 days followed by prednisone 60 mg in a tapering dose RTX 1000 mg i.v. on days 0 and 14	Hashimoto’s thyroiditis Vitiligo Chronic atrophic gastritis MPO–ANCA-associated vasculitis	3

a As reported by the authors.

b Dose and/or frequency not reported.

c No renal biopsy performed.

F: female; M: male; MPA: microscopic polyangiitis; RTX: rituximab.

The pathogenesis of autoimmune polyglandular syndrome remains unclear, but it is most likely to be attributable to a combination of environmental triggers in individuals with genetic susceptibility. Several genes coding for key regulatory proteins in the adaptive and innate immune system, particularly in the MHC, have been associated with autoimmune polyglandular syndrome [2]. In a cohort consisting of Caucasian patients, mostly with autoimmune polyglandular syndrome type 3, HLA class II alleles DRB1*0301, *0401, DQA1*0301, *0501, DQB1*0201 and *0302 were observed more often in autoimmune polyglandular syndrome than in patients with autoimmune thyroid diseases and controls [8]. *HLA-DRB1*, *HLA-DQA1* and *HLA-DQB1* have also been proposed as potential predisposing factors for AAV [9]. Furthermore, several immunoregulatory genes, such as those encoding protein tyrosine phosphatase non-receptor type 22 (PTPN22) and cytotoxic T-lymphocyte-associated antigen 4 (CTLA-4), are associated with an increased risk of autoimmune polyglandular syndrome type 3 and AAV [9, 10]. Polymorphisms rs2476601 (C1858T) of *PTPN22* and rs3087243 (CT60) of *CTLA-4* were found to be associated with autoimmune polyglandular syndrome in Caucasian patients [10]. Interestingly, the same polymorphisms are associated with the occurrence of AAV [9].

We report the rare combination of three well-defined autoimmune diseases (Hashimoto’s thyroiditis, vitiligo and chronic atrophic gastritis) with a severe MPO-AAV in the context of autoimmune polyglandular syndrome type 3 and suggest a pathogenetic link between these diseases. Physicians should be aware that autoimmune polyglandular syndrome increases the risk of the development of other autoimmune components.

## Funding

No specific funding was received from any bodies in the public, commercial or not-for-profit sectors to carry out the work described in this manuscript.

*Disclosure statement:* The authors have declared no conflicts of interest.

## Data Availability

All relevant patient data are included in the paper. Additional data regarding the literature review are available from the corresponding author upon reasonable request.
